# Vaccine Development for Nipah Virus Infection in Pigs

**DOI:** 10.3389/fvets.2019.00016

**Published:** 2019-02-04

**Authors:** Rebecca K. McLean, Simon P. Graham

**Affiliations:** ^1^The Pirbright Institute, Pirbright, United Kingdom; ^2^School of Veterinary Medicine, University of Surrey, Guildford, United Kingdom

**Keywords:** Nipah virus, pigs, zoonosis, epidemiology, pathogenesis, vaccine development

## Abstract

Nipah virus (NiV) causes a severe and often fatal neurological disease in humans. Whilst fruit bats are considered the natural reservoir, NiV also infects pigs and may cause an unapparent or mild disease. Direct pig-to-human transmission was responsible for the first and still most devastating NiV outbreaks in Malaysia and Singapore in 1998–99, with nearly 300 human cases and over 100 fatalities. Pigs can therefore play a key role in the epidemiology of NiV by acting as an “amplifying” host. The outbreak in Singapore ended with the prohibition of pig imports from Malaysia and the Malaysian outbreak was ended by culling 45% of the country's pig population with costs exceeding US$500 million. Despite the importance of NiV as an emerging disease with the potential for pandemic, no vaccines, or therapeutics are currently approved for human or livestock use. In this mini-review, we will discuss current knowledge of NiV infection in pigs; our ongoing work to develop a NiV vaccine for use in pigs; and the pig as a model to support human vaccine development.

## Nipah virus is an Emerging Pathogen With the Potential For Pandemic

Nipah virus (NiV) is an enveloped, single stranded, negative sense RNA paramyxovirus, genus *Henipavirus*. The natural hosts and wildlife reservoirs of NiV are Old World fruit bats of the genus *Pteropus* (1). Both Nipah and the related Hendra virus possess a number of features that distinguish them from other paramyxoviruses. Of particular note is their broad host range which is facilitated by the use of the evolutionary conserved ephrin-B2 and –B3 as cellular receptors (2). The NiV attachment glycoprotein (G) is responsible for binding to ephrin-B2/-B3 (3). Following receptor binding, the G protein dissociates from the fusion (F) protein. Subsequently, the F protein undergoes a series of conformational changes which in turn initiates fusion of the viral and host membrane allowing entry (4). During viral replication, the F protein is synthesized and cleaved into fusion active F1 and F2 subunits. These subunits are subsequently transported back to the cell surface to be incorporated into budding virions, or facilitate fusion between infected and adjacent uninfected cells (5). This cell-to-cell fusion results in the formation of multinucleated cells called syncytia, and greatly influences the cyopathogenicity of NiV as it allows spread of the virus, even in the absence of viral budding (5, 6).

**Figure 1:**
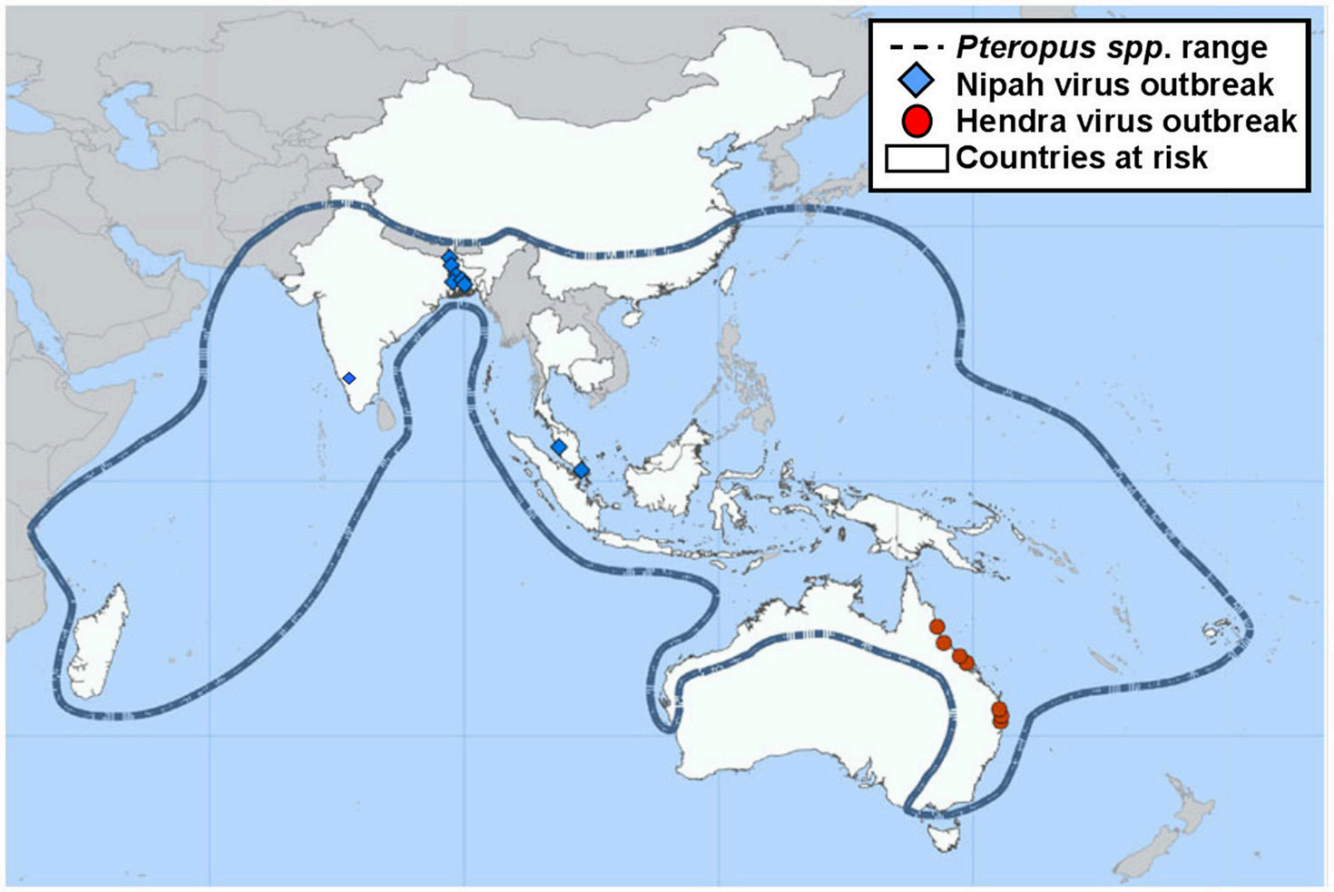
Previous locations of Henipavirus infection outbreaks. Nipah and Hendra virus distribution map highlighting the range of the natural wildlife reservoir, *Pteropus* spp. bats [adapted from (9)].

NiV infection is currently classed as a stage III zoonotic disease, meaning it can spill over to humans and cause limited outbreaks of person-to-person transmission (7, 8). NiV outbreaks have been recognized yearly in Bangladesh since 2001 as well as occasional outbreaks in neighboring India (Figure 1). These outbreaks have been characterized by person-to-person transmission and the death of over 70% of infected people (10, 11). In May 2018, the first ever outbreak in southern India was reported. A total of 19 NiV cases, of which 17 resulted in death, were reported in the state of Kerala. *Pteropus giganteus* bats from areas around the index case in Kozhikode, Kerala, were tested at the National High Security Animal Diseases Laboratory at Bhopal. Of these, 19% were found to be NiV positive by RT-PCR (12). Characteristics of NiV that increase the risk of it becoming a global pandemic include: humans are already susceptible; many NiV strains are capable of person-to-person transmission; and as an RNA virus, NiV has a high mutation rate (13). NiV has been found to survive for up to 4 days when subjected to various environmental conditions, including fruit bat urine and mango flesh (14). Whilst survival time was influenced by fluctuations in both temperature and pH, the ability for NiV to be spread by fomites could play a role in outbreak situations.

The first and still most devastating NiV outbreak occurred in peninsular Malaysia from September 1998 to May 1999 (15, 16). The link to pigs in this outbreak was obvious as 93% of the infected patients had contact with pigs (17). If a NiV strain were to become human-adapted and infect communities in Southeast Asia where there are high human and pig densities and pigs are a primary export commodity, infection could rapidly spread and humanity could face its most devastating pandemic (8, 11, 18).

## The Role of Pigs in the 1998/99 Nipah virus Outbreak

In September 1998, there was an outbreak of severe febrile encephalitis among pig farmers in the state of Perak, Malaysia, that was associated with a high mortality rate. A total of 265 cases of encephalitis, of which 105 resulted in death, were confirmed. These deaths were initially thought to be due to Japanese encephalitis (JE), an endemic disease in Malaysia. However, with most cases occurring in men who worked with pigs, the epidemiological characteristics of this disease were distinct from those of JE, where ~75% of cases occur in children aged 0–14 years (19–21). The epidemiological link was from fruit bats infecting pigs that then served as amplifier hosts, resulting in transmission to humans through close contact (22). As a result of movement of infected pigs and humans to other states in Malaysia, by February 1999 similar diseases were recognized in both pigs and humans in new outbreak areas (23). In the following month, there were 11 cases of respiratory illness and encephalitis amongst Singapore abattoir workers who had handled pigs imported from the outbreak areas in Malaysia (15). Due to this, the importation of pigs from Malaysia ceased which in turn ended the outbreak in Singapore. The outbreak in Malaysia ended when 1.1 million pigs (45% of the country's pig population) were culled from outbreak and surrounding areas (17, 24). The NiV outbreak incurred significant economic costs and long-term damage to the Malaysian pig industry: US$582 million in direct costs and lost market revenue, including US$97 million in compensation to farmers for the 1.1 million pigs slaughtered and 36,000 jobs lost (25). To this date, Malaysian pig farming is only permitted in “identified pig farming areas.”

## Nipah virus Infection in Pigs

Pigs also suffered during the 1998/99 Malaysian outbreak, but this was only diagnosed as part of the investigation following the human cases. The severity of symptoms of NiV infection in pigs varied with age. In suckling pigs (<4 weeks old), mortality could be high (up to 40%) and labored breathing and muscle tremors were evident. In growing pigs (1 to 6 months), an acute febrile (>39.9°C) illness was observed with respiratory signs ranging from increased or forced respiration to a harsh, loud non-productive cough, open mouth breathing, and epistaxis (26). In some cases these respiratory signs were accompanied by one or more of the following neurological signs: trembles, neuralgic twitches, muscle fasciculation, tetanic spasms, incoordination, rear leg weakness, or partial paralysis. Pigs of this age had high morbidity and low mortality (<5%) (26–28). Some animals over 6 months of age died rapidly (within 24 h) without signs of clinical disease. Respiratory signs were reported in adult pigs, as with younger animals, although these were less obvious (labored breathing, bloody nasal discharge, increased salivation) and neurological signs included head pressing, bar biting, tetanic spasms and convulsions. First trimester abortions were also reported (26–28).

In an experimental infection study, pigs were inoculated subcutaneously with a NiV isolate from the central nervous system of a fatally infected human patient. Infection elicited respiratory and neurological symptoms consistent with those observed in naturally infected Malaysian pigs, which included febrile illness, incoordination, mucosal nasal discharge, and persistent cough (29). Pigs inoculated orally with the same dose did not show clinical signs although they still shed virus. In a second study, piglets were inoculated oronasally with a human NiV isolate (30). All infected animals showed a transient increase in body temperature between 4 and 12 days post-infection. Two of these animals developed transient respiratory signs, mild depression and a hunched stance. Both these studies concluded that NiV infection in pigs had no pathognomonic features i.e., the clinical signs observed were non-specific. This can make field diagnosis of NiV infection in pigs difficult, as observed in the outbreak in Malaysia (16, 28).

The name proposed for the disease caused by NiV infection of pigs was “porcine respiratory and neurological syndrome” (also known as “porcine respiratory and encephalitis syndrome”), or, in peninsular Malaysia, “barking pig syndrome” (28). NiV infection was included as the sixth pig disease notifiable to the OIE World Organization for Animal Health (31). The OIE approve diagnostics and recommends preventative and control measures for a range of transboundary livestock diseases.

## Current State of NiV Vaccine Development

Despite the importance of NiV as an emerging disease with the potential for pandemic, no therapeutics or vaccines are approved for use in humans or livestock species. Due to the lethal nature of NiV infection, producing a safe, live attenuated vaccine with no potential for reversion is difficult. However, recombinant NiV mutants, attenuated in hamster and ferret models, have been shown to generate strong neutralizing antibody responses (32, 33). More commonly, NiV vaccine approaches have focused individual candidate antigens delivered as subunit vaccines or using viral vectors. The most studied vaccine candidate is the soluble form of the G protein (sG) from the related Hendra virus (HeV). HeV and the NiV Malaysia strain share between 68 and 92% amino acid homology between their proteins; with F and G proteins sharing 88 and 83% homology, respectively (34). Both F and G envelope glycoproteins are regarded as vaccine candidate antigens since they are the targets of NiV neutralizing antibodies (35).

An adjuvanted HeV sG protein subunit-based vaccine (Equivac® HeV, Zoetis) has been licensed in Australia to protect horses against HeV and to reduce the zoonotic risk to humans (36). Equivac® HeV protects ferrets and African green monkeys (AGMs) after experimental challenge with NiV, as well as HeV (37, 38). Surprisingly, this vaccine failed to protect pigs from experimental NiV challenge (39). Since the vaccine induced cross-neutralizing antibodies but not measurable T cell responses, the authors concluded that both arms of the adaptive immune response may be required for protection against NiV and HeV. These studies also potentially highlight that adjuvants can have species specific effects and tailoring of adjuvants to the target species may be required or considered in the context of preclinical models. The experimental viral vectored vaccine candidates for NiV include vesicular stomatitis virus, rabies virus, canarypox virus (ALVAC strain), adeno-associated virus (AAV), measles virus, Newcastle disease virus (NDV) and Venezuelan equine encephalitis virus (40). ALVAC expressing NiV G or F (ALVAC-G and ALVAC-F) was found to protect pigs against NiV challenge 2 weeks after the second immunization (41). High titres of NiV neutralizing antibodies were induced with the ALVAC-G vaccine, while despite the low levels of neutralizing antibodies induced by the ALVAC-F; all vaccinated pigs were protected against virulent NiV challenge. Recombinant attenuated NDV expressing NiV glycoproteins have been shown to induce long lasting NiV-specific nAbs in pigs, with the vector expressing NiV G performing better than NiV F (42). However, no challenge was performed in this study and it remains to be determined whether these paramyxovirus-based vaccine candidates are efficacious. Compared to canarypox vectors, NDV-based vectors have a number of advantages including their high titer propagation in chicken eggs removing the requirement for cell culture (41, 42). Despite these encouraging results and the continued threat posed by NiV, no vaccine candidate has progressed toward market for either pigs or humans.

## The Development of a NiV Vaccine for Pigs

The promising performance of experimental NiV and HeV vaccines in animal models and the licensure of Equivac® HeV, as a “One Health” vaccine to safeguard animal and human health, strongly support the proposition that a safe and effective NiV vaccine may be developed for pigs to reduce the severe economic consequences of NiV outbreaks and the threat to public health. With partners, we have initiated a project that aims to develop such a vaccine. We are systematically analyzing the immunogenicity and protective efficacy of three NiV vaccine candidates in pigs: (1) an adjuvanted NiV sG protein (orthologous to the Equivac® HeV vaccine), (2) NiV G protein delivered by a replication-deficient simian adenoviral vector (ChAdOx1 NiV G), and an adjuvanted, molecular clamp stabilized NiV F (mcsF) protein. ChAdOx1 is a multispecies vector with an established human and livestock safety profile (43). ChAdOx1 offers the potential for both single dose efficacy and thermostabilization (44, 45). The molecular clamp is a proprietary stabilization domain that preserves the F protein in its native “pre-fusion” form, which should enhance immunogenicity and thermostability. In depth analyses of T cell and antibody responses are being conducted to identify correlates of vaccine-induced protection. We will examine the durability of NiV-neutralizing antibodies and other immune responses associated with protection, including a comparison of a single-shot vs. homologous prime-boost immunization regimes. In-contact animals will be introduced to assess transmission of challenge virus from vaccinates or unvaccinated control animals.

The sporadic nature of NiV outbreaks means that the commercial development of NiV vaccines for use in pigs (other livestock or humans) is limited and animal health companies are of the opinion that NiV vaccines will have limited marketability. Our ongoing studies should help facilitate this by developing a safe and efficacious prototype NiV vaccine that is amenable to “surge production” and discrimination of infection in vaccinated animals (DIVA) capability. Subsequent development and licensure of this vaccine will require engagement with international, regional, and national agencies and the creation of dependable markets via the establishment of NiV vaccine banks. The OIE World Fund manages vaccine banks and the delivery of vaccines for avian influenza, rabies, foot-and-mouth disease, and peste de petit ruminants (46). Vaccine banks ensure the procurement and delivery of high quality vaccines mass-produced in line with OIE intergovernmental standards. Critically these vaccine banks can be rapidly deployed when required and this model appears most appropriate in the context of reactive emergency vaccination programmes to aid NiV outbreak control. Vaccines can play a major component in an emergency response against emerging infectious disease, with the main aim to reduce virus spread between susceptible hosts (47). The precise decisions on control strategies will be complex and vary for different regions. Factors such as: herd density, production systems, the presence of susceptible wildlife, the impact on export trade and current opinions on economic vs. ethical factors will likely play a role. One strategy to halt a NiV outbreak would be to deploy a stockpiled vaccine for ring vaccination around the NiV affected area. This approach was utilized in the 2016 Ebola outbreak in Guinea and showed great promise in terms of disease containment and elimination (48). For such a strategy, a vaccine with single-dose efficacy and a rapid onset of immunity preventing virus transmission would be preferential. This is likely to be best achieved with a viral-vectored (45) or mRNA vectored vaccine (49). The highly unpredictable nature of NiV outbreaks means that it is highly unlikely that NiV vaccines would be used routinely by pig producers. One strategy that could help ensure that immunity to NiV is maintained in pig herds could involve the engineering of NiV G into a live attenuated viral vaccine, such as pseudorabies, which are widely used in countries at-risk.

## The pig as a Model for Human NiV

The recent Ebola and Zika epidemics highlighted how poorly prepared we were to deal with these new and emerging diseases. There has therefore been a global drive to develop vaccines against these diseases and improve preparedness. The Coalition for Epidemic Preparedness Innovation's (CEPI's) was established in 2016 with a mandate of financing and coordinating the development of new human vaccines to prevent and contain infectious disease epidemics. CEPI selected NiV, Lassa virus and Middle East respiratory syndrome-coronavirus, three pathogens from the WHO's list of priority diseases needing urgent R&D attention as its initial focus (50, 51). The WHO's list of priority diseases is part of the R&D Blueprint, which identifies priority diseases and addresses gaps in the global scientific community to increase preparedness for future outbreaks. The main aim of the Blueprint is to fast-track the availability of effective tests, vaccines, and medicines that can be used to save lives and avert large scale crises (51).

In 2002, the US Food and Drug Administration (FDA) established the “Animal Rule” for regulatory approval of vaccines and therapeutics for which efficacy testing in humans is impossible, therefore requiring relevant animal models that represent a disease model similar to that of the human disease (52). Vaccine efficacy studies in animal models aim to identify specific vaccine-induced correlates of protection including neutralizing antibodies or cell-mediated responses (53). In 2015, a vaccine to protect against anthrax was the first to be approved through the “animal rule” (54). The licensing pathway for the “Animal Rule” requires that immunogenicity results from clinical trials must be consistent with previously identified immune correlates associated with protection (52). Therefore, identifying reliable markers of vaccine-generated immunity becomes critically important for pathogens such as NiV. Large animal models have been shown to more accurately predict vaccine outcome in humans in comparison to small animal models (55) therefore defining correlates of vaccine-induced protection in pigs, may play an important role in supporting subsequent human vaccine licensure under the “Animal Rule.”

Animal models can be validated for a particular disease according to a number of different criteria, which include “face” and “predictive” validity. For face validity there must be similarities in the pathology and clinical symptoms between the animal model and the human disease (56). As discussed above, NiV infection of pigs causes a similar respiratory and neurological syndrome as seen in human infections. Although, disease severity in pigs may be considered lower than in humans. The predictive validity of a model means that clinically effective interventions demonstrate a similar effect in the animal model (56). No clinical trials of NiV vaccine candidates have been reported to compare with vaccine performance in animal models, including the pig. As noted above, the success of the Equivac® HeV vaccine in horses and other animal models was not replicated in swine (38, 39), highlighting a potential issue of predicative validity when comparing NiV vaccines between animal species, which may extend to humans. On the other hand, pigs have been used successfully as models to study many human infectious diseases (57–63), including NiV infection (64). There is also a growing appreciation that pigs provide a superior animal model for influenza A virus infection and immunity and should play a more prominent role as a model for human influenza vaccine development (65). The success of the pig as an experimental animal model is partly due to their similarities with humans in terms of anatomy, immunology, and physiology, but also due to their manageable behavior and size, and by the general ethical acceptance of using pigs for experimental purposes instead of non-human primates (55, 63, 66).

## Conclusions

The NiV outbreaks in Malaysia and Singapore demonstrated that pigs can play a key role in the epidemiology of NiV by acting as an amplifier host. The region most at risk of NiV infection has some of the highest pig population densities found anywhere in the world, which are rising fast due to the demand of a growing human population. This increases the risk of NiV transmission to pigs and humans. The development of a NiV vaccine for use in pig populations would decrease the major risk NiV poses to the developing pig industries, as well as to the livelihoods of poor livestock keepers in Southeast Asia. The use of non-human animal models is crucial for vaccine development against diseases such as NiV since efficacy testing in humans is impossible. The pig model may therefore contribute to human vaccine development, supporting human vaccine licensure under the Animal Rule.

## Author Contributions

All authors listed have made a substantial, direct and intellectual contribution to the work, and approved it for publication.

### Conflict of Interest Statement

The authors declare that the research was conducted in the absence of any commercial or financial relationships that could be construed as a potential conflict of interest.

## References

[1] Halpin K Hyatt AD Fogarty R Middleton D Bingham J Epstein JH. Pteropid bats are confirmed as the reservoir hosts of Henipaviruses: a comprehensive experimental study of virus transmission. Am J Trop Med Hyg. (2011) 85:946–51. 10.4269/ajtmh.2011.10-0567 22049055 PMC3205647

[2] Bossart KN Tachedjian M McEachern JA Crameri G Zhu Z Dimitrov DS. Functional studies of host-specific ephrin-B ligands as Henipavirus receptors. Virology (2008) 372:357–71. 10.1016/j.virol.2007.11.011 18054977

[3] Bowden TA Aricescu AR Gilbert RJ Grimes JM Jones EY Stuart DI. Structural basis of Nipah and Hendra virus attachment to their cell-surface receptor ephrin-B2. Nat Struct Mol Biol. (2008) 15:567–72. 10.1038/nsmb.1435 18488039

[4] Liu Q Stone JA Bradel-Tretheway B Dabundo J Benavides Montano JA. Unraveling a three-step spatiotemporal mechanism of triggering of receptor-induced Nipah virus fusion and cell entry. PLoS Pathog. (2013) 9:e1003770. 10.1371/journal.ppat.1003770 24278018 PMC3837712

[5] Maisner A Neufeld J Weingartl H. Organ- and endotheliotropism of Nipah virus infections *in vivo* and *in vitro*. Thromb Haemost. (2009) 102:1014–23. 10.1160/th09-05-0310 19967130

[6] Diederich S Thiel L Maisner A. Role of endocytosis and cathepsin-mediated activation in Nipah virus entry. Virology (2008) 375:391–400. 10.1016/j.virol.2008.02.019 18342904 PMC7103400

[7] Wolfe ND Dunavan CP Diamond J. Origins of major human infectious diseases. Nature (2007) 447:279–83. 10.1038/nature05775 17507975 PMC7095142

[8] Luby SP. The pandemic potential of Nipah virus. Antiviral Res. (2013) 100:38–43. 10.1016/j.antiviral.2013.07.011 23911335

[9] CDC Nipah Virus Distribution Map [Online]. (2014). Available online at: https://www.cdc.gov/vhf/nipah/outbreaks/distribution-map.html (Accessed August 21, 2018).

[10] Hsu VP Hossain MJ Parashar UD Ali MM Ksiazek TG Kuzmin I. Nipah virus encephalitis reemergence, Bangladesh. Emerging Infect Dis. (2004) 10:2082–7. 10.3201/eid1012.040701 15663842 PMC3323384

[11] Donaldson H Lucey D. Enhancing preparation for large Nipah outbreaks beyond Bangladesh: preventing a tragedy like Ebola in West Africa. Int J Infect Dis. (2018) 72:69–72. 10.1016/j.ijid.2018.05.015 29879523 PMC7110759

[12] WHO Nipah Virus - India: Disease Outbreak News [Online]. (2018). Available online at: http://www.who.int/csr/don/07-august-2018-nipah-virus-india/en/ (Accessed August 23, 2018).

[13] Kulkarni S Volchkova V Basler CF Palese P Volchkov VE Shaw ML. Nipah virus edits its P gene at high frequency to express the V and W proteins. J Virol. (2009) 83:3982–7. 10.1128/JVI.02599-08 19211754 PMC2663244

[14] Fogarty R Halpin K Hyatt AD Daszak P Mungall BA. Henipavirus susceptibility to environmental variables. Virus Res. (2008) 132:140–4. 10.1016/j.virusres.2007.11.010 18166242 PMC3610175

[15] Paton NI Leo YS Zaki SR Auchus AP Lee KE Ling AE. Outbreak of Nipah-virus infection among abattoir workers in Singapore. Lancet (1999) 354:1253–6. 10.1016/s0140-6736(99)04379-2 10520634

[16] Chua KB. Nipah virus outbreak in Malaysia. J Clin Virol. (2003) 26:265–75. 12637075 10.1016/s1386-6532(02)00268-8

[17] Parashar UD Sunn LM Ong F Mounts AW Arif MT Ksiazek TG. Case-control study of risk factors for human infection with a new zoonotic paramyxovirus, Nipah virus, during a 1998-1999 outbreak of severe encephalitis in Malaysia. J Infect Dis. (2000) 181:1755–9. 10.1086/315457 10823779

[18] Huynh TT Aarnink AJ Drucker A Verstegen MW Pig production in Cambodia, Laos, Philippines and Vietnam: a review. Asian J Agric Dev. (2007) 1:323–39.

[19] Chua KB Bellini WJ Rota PA Harcourt BH Tamin A Lam SK. Nipah virus: a recently emergent deadly Paramyxovirus. Science (2000) 288:1432–5. 10.1126/science.288.5470.1432 10827955

[20] Lam SK Chua KB. Nipah virus encephalitis outbreak in Malaysia. Clin Infect Dis. (2002) 34(Suppl. 2):S48–51. 10.1086/338818 11938496

[21] Malhotra S Sharma S Hans C Japanese Encephalitis and its epidemiology. J Infect Dis Ther. (2015) 3:243 10.4172/2332-0877.1000243

[22] Daszak P Zambrana-Torrelio C Bogich TL Fernandez M Epstein JH Murray KA. Interdisciplinary approaches to understanding disease emergence: the past, present, and future drivers of Nipah virus emergence. Proc Natl Acad Sci USA. (2013) 110(Suppl. 1):3681–8. 10.1073/pnas.1201243109 22936052 PMC3586606

[23] CDC Outbreak of Hendra-Like Virus - Malaysia and Singapore, 1998-1999 [Online]. (1999). Available online at: https://www.cdc.gov/mmwr/preview/mmwrhtml/00056866.htm (Accessed August 21, 2018).

[24] Enserink M. New virus fingered in malaysian epidemic. Science (1999) 284:407–10. 10.1126/science.284.5413.407 10232977

[25] Dimmock NJ Easton AJ Leppard KN Introduction to Modern Virology. Hoboken, NJ: Blackwell Publishing Ltd (2016).

[26] OIE Nipah Virus [Online]. (2018). Available online at: http://www.oie.int/en/animal-health-in-the-world/animal-diseases/Nipah-Virus/ (Accessed September 27, 2018).

[27] Aziz AJ Mahendran R Daniels P Shahiruddin S Narasiman M Azizah D The status, public response and challenges in overcoming emerging and exotic diseases - Nipah virus disease experience. In: National Congress on Animal Health and Production: Environmental Care in Animal Production. (Alor Gajah) (1999).

[28] Nor MNM Gan CH Ong BL Nipah virus infection of pigs in peninsular Malaysia. Rev Off Int Epizoot. (2000) 19:160–5. 10.20506/rst.19.1.1202 11189713

[29] Middleton DJ Westbury HA Morrissy CJ van der Heide BM Russell GM Braun MA. Experimental Nipah virus infection in pigs and cats. J Comp Pathol. (2002) 126:124–36. 10.1053/jcpa.2001.0532 11945001

[30] Berhane Y Weingartl HM Lopez J Neufeld J Czub S Embury-Hyatt C. Bacterial infections in pigs experimentally infected with Nipah virus. Transbound Emerg Dis. (2008) 55:165–74. 10.1111/j.1865-1682.2008.01021.x 18405339

[31] OIE OIE-Listed Diseases, Infections and Infestations in Force in 2018 [Online]. (2018). Available online at: http://www.oie.int/animal-health-in-the-world/oie-listed-diseases-2018/ (Accessed September 27, 2018).

[32] Yoneda M Guillaume V Sato H Fujita K Georges-Courbot MC Ikeda F. The nonstructural proteins of Nipah virus play a key role in pathogenicity in experimentally infected animals. PLoS ONE (2010) 5:e12709. 10.1371/journal.pone.0012709 20856799 PMC2939873

[33] Satterfield BA Cross RW Fenton KA Agans KN Basler CF Geisbert TW. The immunomodulating V and W proteins of Nipah virus determine disease course. Nat Commun. (2015) 6:7483. 10.1038/ncomms8483 26105519 PMC4482017

[34] Harcourt BH Tamin A Ksiazek TG Rollin PE Anderson LJ Bellini WJ. Molecular characterization of Nipah virus, a newly emergent paramyxovirus. Virology (2000) 271:334–49. 10.1006/viro.2000.0340 10860887

[35] Broder CC Xu K Nikolov DB Zhu Z Dimitrov DS Middleton D. A treatment for and vaccine against the deadly Hendra and Nipah viruses. Antiviral Res. (2013) 100:8–13. 10.1016/j.antiviral.2013.06.012 23838047 PMC4418552

[36] Middleton D Pallister J Klein R Feng YR Haining J Arkinstall R. Hendra virus vaccine, a one health approach to protecting horse, human, and environmental health. Emerging Infect Dis. (2014) 20:372–9. 10.3201/eid2003.131159 24572697 PMC3944873

[37] Pallister J Middleton D Wang LF Klein R Haining J Robinson R. A recombinant Hendra virus G glycoprotein-based subunit vaccine protects ferrets from lethal hendra virus challenge. Vaccine (2011) 29:5623–30. 10.1016/j.vaccine.2011.06.015 21689706 PMC3153950

[38] Mire CE Geisbert JB Agans KN Feng YR Fenton KA Bossart KN. A recombinant Hendra virus G glycoprotein subunit vaccine protects nonhuman primates against Hendra virus challenge. J Virol. (2014) 88:4624–31. 10.1128/JVI.00005-14 24522928 PMC3993805

[39] Pickering BS Hardham JM Smith G Weingartl ET Dominowski PJ Foss DL. Protection against henipaviruses in swine requires both, cell-mediated and humoral immune response. Vaccine (2016) 34:4777–86. 10.1016/j.vaccine.2016.08.028 27544586 PMC6161494

[40] Satterfield BA Dawes BE Milligan GN. Status of vaccine research and development of vaccines for Nipah virus. Vaccine (2016) 34:2971–5. 10.1016/j.vaccine.2015.12.075 26973068

[41] Weingartl HM Berhane Y Caswell JL Loosmore S Audonnet JC Roth JA. Recombinant nipah virus vaccines protect pigs against challenge. J Virol. (2006) 80:7929–38. 10.1128/jvi.00263-06 16873250 PMC1563797

[42] Kong D Wen Z Su H Ge J Chen W Wang X. Newcastle disease virus-vectored Nipah encephalitis vaccines induce B and T cell responses in mice and long-lasting neutralizing antibodies in pigs. Virology (2012) 432:327–35. 10.1016/j.virol.2012.06.001 22726244

[43] Dicks MD Spencer AJ Edwards NJ Wadell G Bojang K Gilbert SC. A novel chimpanzee adenovirus vector with low human seroprevalence: improved systems for vector derivation and comparative immunogenicity. PLoS ONE (2012) 7:e40385. 10.1371/journal.pone.0040385 22808149 PMC3396660

[44] Dulal P Wright D Ashfield R Hill AV Charleston B Warimwe GM. Potency of a thermostabilised chimpanzee adenovirus Rift Valley Fever vaccine in cattle. Vaccine (2016) 34:2296–8. 10.1016/j.vaccine.2016.03.061 27020712 PMC4851241

[45] Warimwe GM Gesharisha J Carr BV Otieno S Otingah K Wright D. Chimpanzee adenovirus vaccine provides multispecies protection against Rift Valley Fever. Sci Rep. (2016) 6:20617. 10.1038/srep20617 26847478 PMC4742904

[46] OIE Vaccine Banks [Online]. (2018). Available online at: http://www.oie.int/en/solidarity/vaccine-banks/ (Accessed September 27, 2018).

[47] Nii-Trebi NI. Emerging and neglected infectious diseases: insights, advances, and challenges. Biomed Res Int. (2017) 2017:5245021. 10.1155/2017/5245021 28286767 PMC5327784

[48] Henao-Restrepo AM Longini IM Egger M Dean NE Edmunds WJ Camacho A. Efficacy and effectiveness of an rVSV-vectored vaccine expressing Ebola surface glycoprotein: interim results from the Guinea ring vaccination cluster-randomised trial. Lancet (2015) 386:857–66. 10.1016/S0140-6736(15)61117-5 26248676

[49] Pardi N Hogan MJ Pelc RS Muramatsu H Andersen H DeMaso CR. Zika virus protection by a single low-dose nucleoside-modified mRNA vaccination. Nature (2017) 543:248–51. 10.1038/nature21428 28151488 PMC5344708

[50] CEPI Priority Diseases [Online]. (2018). Available online at: http://cepi.net/resources (Accessed September 27, 2018).

[51] WHO A Research and Development Blueprint for Action to Prevent Epidemics [Online]. (2018). Available online at: http://www.who.int/blueprint/en/ (Accessed September 27, 2018).

[52] FDA New drug and biological drug products; evidence needed to demonstrate effectiveness of new drugs when human efficacy studies are not ethical or feasible. Final rule. Fed Regist. (2002) 67:37988–98. 12049094

[53] Meyer M Malherbe DC Bukreyev A. Can Ebola virus vaccines have universal immune correlates of protection? Trends Microbiol. (2018). 27:8–16. 10.1016/j.tim.2018.08.008 30201511 PMC6309495

[54] Beasley DWC Brasel TL Comer JE. First vaccine approval under the FDA Animal Rule. NPJ Vaccines (2016) 1:16013. 10.1038/npjvaccines.2016.13 29263855 PMC5707879

[55] Gerdts V Wilson HL Meurens F van Drunen Littel-van den Hurk S Wilson D Walker S. Large animal models for vaccine development and testing. ILAR J (2015) 56:53–62. 10.1093/ilar/ilv009 25991698

[56] Denayer T Stöhr T Van Roy M Animal models in translational medicine: validation and prediction. NHTM (2014) 2:5–11. 10.1016/j.nhtm.2014.08.001

[57] Svedman P Ljungh A Rausing A Banck G Sanden G Miedzobrodzki J Staphylococcal wound infection in the pig: Part I. Course Ann Plast Surg. (1989) 23:212–8. 2782820 10.1097/00000637-198909000-00004

[58] He YG McCulley JP Alizadeh H Pidherney M Mellon J Ubelaker JE. A pig model of Acanthamoeba keratitis: transmission via contaminated contact lenses. Invest Ophthalmol Vis Sci. (1992) 33:126–33. 1730533

[59] Nedrud JG. Animal models for gastric Helicobacter immunology and vaccine studies. FEMS Immunol Med Microbiol. (1999) 24:243–50. 10.1111/j.1574-695X.1999.tb01290.x 10378428

[60] Elahi S Holmstrom J Gerdts V. The benefits of using diverse animal models for studying pertussis. Trends Microbiol. (2007) 15:462–8. 10.1016/j.tim.2007.09.003 17920273

[61] Luna CM Sibila O Agusti C Torres A. Animal models of ventilator-associated pneumonia. Eur Respir J. (2009) 33:182–8. 10.1183/09031936.00046308 19118229

[62] Khatri M Dwivedi V Krakowka S Manickam C Ali A Wang LF. Swine influenza H1N1 virus induces acute inflammatory immune responses in pig lungs: a potential animal model for human H1N1 influenza virus. J Virol. (2010) 84:11210–8. 10.1128/jvi.01211-10 20719941 PMC2953174

[63] Meurens F Summerfield A Nauwynck H Saif L Gerdts V. The pig: a model for human infectious diseases. Trends Microbiol. (2012) 20:50–7. 10.1016/j.tim.2011.11.002 22153753 PMC7173122

[64] Weingartl HM Berhane Y Czub M. Animal models of henipavirus infection: a review. Vet J. (2009) 181:211–20. 10.1016/j.tvjl.2008.10.016 19084436

[65] Rajao DS Vincent AL. Swine as a model for influenza A virus infection and immunity. ILAR J. (2015) 56:44–52. 10.1093/ilar/ilv002 25991697

[66] Käser T Renois F Wilson HL Cnudde T Gerdts V Dillon JR. Contribution of the swine model in the study of human sexually transmitted infections. Infect. Genet. Evol. (2018) 66:346–60. 10.1016/j.meegid.2017.11.022 29175001

